# Adapting inpatient addiction medicine consult services during the COVID-19 pandemic

**DOI:** 10.1186/s13722-021-00221-1

**Published:** 2021-02-24

**Authors:** Miriam T. H. Harris, Alyssa Peterkin, Paxton Bach, Honora Englander, Emily Lapidus, Theresa Rolley, Melissa B. Weimer, Zoe M. Weinstein

**Affiliations:** 1 Grayken Center for Addiction, Boston Medical Center, Boston, MA USA; 2 Clinical Addiction Research and Education (CARE) Unit, Section of General Internal Medicine, Department of Medicine, Boston University School of Medicine and Boston Medical Center, 801 Massachusetts Avenue, Second Floor, Boston, MA 02118 USA; 3 British Columbia Centre on Substance Use, St. Paul’s Hospital, Vancouver, BC Canada; 4 Department of Medicine, University of British Columbia, Vancouver, BC Canada; 5 Division of Hospital Medicine, Department of Medicine, Oregon Health and Science University, Portland, OR USA; 6 Program in Addiction Medicine, Department of Medicine, Yale School of Medicine, New Haven, CT USA

**Keywords:** Addiction, Inpatient, Consult, COVID-19, Substance use disorders, Homelessness, COVID-19, Social-distancing

## Abstract

**Background:**

We describe addiction consult services (ACS) adaptations implemented during the Novel Coronavirus Disease 2019 (COVID-19) pandemic across four different North American sites: St. Paul’s Hospital in Vancouver, British Columbia; Oregon Health & Sciences University in Portland, Oregon; Boston Medical Center in Boston, Massachusetts; and Yale New Haven Hospital in New Haven, Connecticut.

**Experiences:**

ACS made system, treatment, harm reduction, and discharge planning adaptations. System changes included patient visits shifting to primarily telephone-based consultations and ACS leading regional COVID-19 emergency response efforts such as substance use treatment care coordination for people experiencing homelessness in COVID-19 isolation units and regional substance use treatment initiatives. Treatment adaptations included providing longer buprenorphine bridge prescriptions at discharge with telemedicine follow-up appointments and completing benzodiazepine tapers or benzodiazepine alternatives for people with alcohol use disorder who could safely detoxify in outpatient settings. We believe that regulatory changes to buprenorphine, and in Vancouver other medications for opioid use disorder, helped increase engagement for hospitalized patients, as many of the barriers preventing them from accessing care on an ongoing basis were reduced. COVID-19 specific harm reductions recommendations were adopted and disseminated to inpatients. Discharge planning changes included peer mentors and social workers increasing hospital in-reach and discharge outreach for high-risk patients, in some cases providing prepaid cell phones for patients without phones.

**Recommendations for the future:**

We believe that ACS were essential to hospitals’ readiness to support patients that have been systematically marginilized during the pandemic. We suggest that hospitals invest in telehealth infrastructure within the hospital, and consider cellphone donations for people without cellphones, to help maintain access to care for vulnerable patients. In addition, we recommend hospital systems evaluate the impact of such interventions. As the economic strain on the healthcare system from COVID-19 threatens the very existence of ACS, overdose deaths continue rising across North America, highlighting the essential nature of these services. We believe it is imperative that health care systems continue investing in hospital-based ACS during public health crises. 
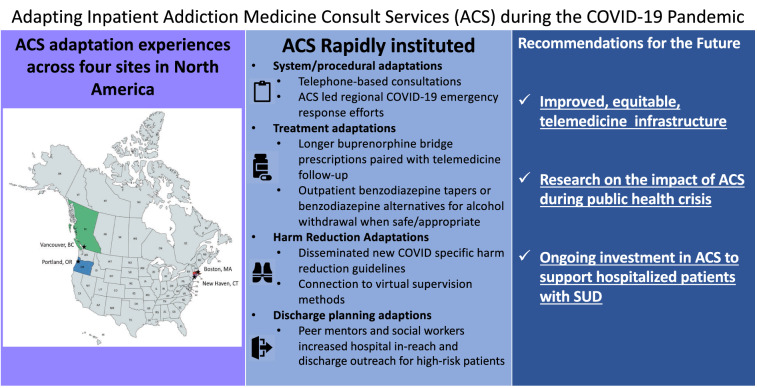

## Introduction

The Novel Coronavirus Disease 2019 (COVID-19) pandemic forced hospital inpatient addiction consult services (ACS) to rapidly evolve as hospitals modified operations to care for people with COVID-19 and protect staff and other patients from infection. When hospital systems are overwhelmed there is a risk that people with substance use disorders (SUD) will be further deprioritized as well-known structural barriers to treatment, namely stigma, lack of clinician training in addiction, and clinician discomfort, may be exacerbated [1].

It is critical to continue to prioritize and address substance use in the hospital setting as people with SUD may be particularly vulnerable to the psychosocial and medical effects of the COVID-19 pandemic. People with SUD may be at increased risk of relapse and acquiring infections such as HIV and HCV due to social distancing mandates causing isolation and stress, as well as disruptions to harm reduction and treatment services. Additionally, factors including homelessness and increased rates of incarceration place people with SUD at higher risk of contracting COVID-19, and higher rates of medical comorbidities also increases their risk of severe COVID-19 infection [2]. Alarmingly, opioid overdose rates in 2020 have already exceeded those of other years and continue to rise [3]. For all of these reasons, hospital-based addiction care, which has been shown to reduce substance use [1], improve patient trust in clinicians [1], increase addiction treatment retention [1], and reduce recurrent hospitalizations [4], remains of utmost importance during the COVID-19 pandemic.

COVID-19 introduces challenges and some potential opportunities in caring for hospitalized people with SUD. In this perspective piece, we describe real world ACS adaptation experiences from: St. Paul’s Hospital in Vancouver, British Columbia; Oregon Health & Sciences University (OHSU) in Portland, Oregon; Boston Medical Center (BMC) in Boston, Massachusetts; and Yale New Haven Hospital (YNHH) in New Haven, Connecticut instituted during the COVID-19 pandemic. We reflect on our experiences to inform ACS responses during this current and for future public health emergencies and highlight areas in need of research.

### Hospital-based addiction consult service adaptations

#### ACS adaptations context and timing

In British Columbia, the first case of COVID-19 was reported on January 28th, 2020. Increasingly strict physical distancing regulations were implemented in the following months, but due to generally low overall provincial case counts hospital personnel continued to deliver in person care at St. Paul’s throughout the pandemic. Oregon confirmed its first case of COVID-19 on February 28th, and by March 13th OHSU had implemented COVID-19 modified operations including restricting all patient visitors and encouraging staff who could to utilize telemedicine to preserve personal protective equipment (PPE) and promote physical distancing. In Massachusetts the first case of COVID-19 was reported on February 1st, and in March BMC rapidly reorganized by shifting outpatient care to predominately telemedicine and recommending that inpatient hospital staff when possible work from home with patient visits being conducted by phone to similarly conserve PPE and protect staff from infection. Connecticut confirmed its first case of COVID-19 on March 8th, with comparable rapid reorganization occurring at YNHH in March to support PPE conservation and infection control.

Policy changes to medications for opioid use disorder (MOUD) access and administration were rapidly enacted in all settings to help fight the spread of COVID-19. In British Columbia increased flexibility for patients accessing MOUD were first recommended on March 17th, 2020, with interim clinical guidance on the prescribing of pharmaceutical alternatives to illicit opioids (“safe supply”) issued shortly afterwards [5]. Such policies were gradually adopted into the work of the ACS team at St. Paul’s Hospital over the year in response to rising case numbers and worsening of the overlapping overdose crisis. Following the public health state of emergency declaration in the United Sates (US) on January 31st, 2020 the Acting Drug Enforcement Administration and the Substance Abuse and Mental Health Service Administration stipulated in March 2020 that telemedicine could be used to initiate buprenorphine [6] and they developed new methadone opioid treatment program guidelines [7] that liberalized take home methadone dosing. ACS leadership at OHSU, BMC, and YNHH all quickly instituted ACS adaptations starting in March 2020 that incorporated these policy changes and hospital wide infection control and PPE conservation efforts into new workflows, billing requirements, and modes of patient and team communication.

### System & procedural adaptations

Across most of our respective ACS, early in the pandemic, patient visits shifted to primarily telephone-based consultation paired with ACS staff working from home to conserve PPE and promote physical distancing (please see Table 1 for all site-specific adaptations). However, both system level issues as well as patient-specific factors necessitated in-person contact at times. Across all sites hospital room phones were often broken or absent and many patients did not have working cell phones, obliging in-person visits. Video capability was not yet established at the four ACS sites nor feasible in the beginning of the COVID-19 pandemic. Telephone visits also limited clinicians’ access to non-verbal information, therefore, in-person visits were helpful for patients with limited engagement, acute pain, somnolence or acute intoxication.

**Table 1:**
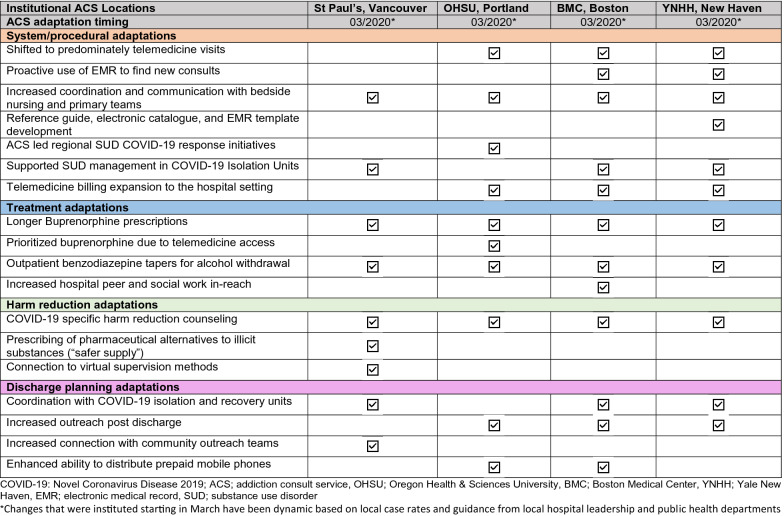
Incremental cost utility ratio (QALY obtained from Index Value)

COVID-19: Novel Coronavirus Disease 2019; ACS; addiction consult service, OHSU; Oregon Health & Sciences University, BMC; Boston Medical Center, YNHH; Yale New Haven, EMR; electronic medical record, SUD; substance use disorder

*Changes that were instituted starting in March have been dynamic based on local case rates and guidance from local hospital leadership and public health departments

ACS teams were concerned that the reduced patient contact and visitation limitations would make hospitalization experiences more stressful. Therefore, at BMC, when possible, peer mentors increased contact with patients through daily tele-check ins. At all sites, ACS also enhanced their relationships with other care providers, like the bedside nurse and primary team, who took over delivering wellness materials such as, art supplies, journaling resources, and readings in addition to providing clinical updates regarding management of withdrawal and safety assessments to ACS teams.

At BMC and YNHH, during the initial peak of regional COVID-19 cases, ACS consult requests declined despite high overall hospital volumes. Because early engagement is critical to treat acute substance withdrawal, initiate effective SUD treatments, and reduce the risk of leaving prior to medical treatment completion [1], ACS teams at BMC and YNHH pursued proactive outreach by contacting primary teams where SUD was noted in patients’ problem lists, or where buprenorphine and methadone were prescribed, to address SUD and support therapeutic alliances between patients and primary teams [1].

ACS led and adopted hospital and regional initiatives in response to SUD systems’ disruptions. The YNHH ACS team developed reference guides [8], and OHSU, BMC, and YNHH created electronic catalogues, and electronic medical record templates that were constantly updated to reflect the rapidly evolving community treatment landscape. These were shared with patients and other primary hospital teams. OHSU ACS leadership convened community SUD leaders through a SUD COVID-19 Response ECHO tele-mentoring program to support an integrated, collaborative pandemic response, partly to support community treatment access statewide. At BMC, in order to minimize readmissions, ACS proactively offered enhanced peer outreach and treatment system navigation to patients after discharge.

### Treatment adaptations

Regulatory changes in the US and Canada [5–7, 9] instituted to adapt outpatient treatment of SUD to the COVID-19 pandemic had perceived impacts on our inpatient addiction treatment experiences. For example, for patients continued or initiated on buprenorphine in the hospital, clinicians commonly paired longer buprenorphine bridge prescriptions at discharge with telemedicine follow-up appointments [9]. At OHSU, this flexibility was associated with perceived increased interest in buprenorphine and engagement from hospitalized patients. At BMC, a local opioid treatment program with close hospital ties relaxed intake procedures regarding the type of photo identification accepted for intake, which we believe resulted in methadone access expansion for inpatients. To mitigate interruptions in methadone treatment in patients who were infected with COVID-19, YNHH worked with local opioid treatment programs to facilitate delivery of methadone to patient’s homes or other treatment settings made possible by the liberalization to take home methadone. In Vancouver, Canada, restrictions were relaxed on take-home opioid agonist treatments (methadone, buprenorphine, and slow-release oral morphine) and in select cases physicians at St. Paul’s hospital were encouraged to consider prescribing pharmaceutical alternatives to illicit substances (i.e. “safe supply” e.g. prescribed hydromorphone tablets for consumption and not treatment of OUD) in order to help promote physical distancing recommendations [5]. In St. Paul’s, ACS felt that these changes helped increase engagement for hospitalized patients, as many of the barriers preventing them from accessing care on an ongoing basis were reduced.

People with alcohol use disorder requested more outpatient detoxification, rather than traditional placement to a detoxification facility or residential treatment program. Therefore, across most sites ACS recommended completing benzodiazepine tapers or benzodiazepine alternatives for patients who could safely detoxify in the outpatient setting. Outpatient detoxification were only completed if patients initially requiring hospitalization for alcohol withdrawal were clinically stable at discharge [9], had safe place to complete the detoxification, and had an outpatient clinician who could complete a follow up assessment.

### Harm reduction adaptations

COVID-19 challenged traditional inpatient harm reduction recommendations and supports. Messages like “don’t use alone” contradicted new physical distancing mandates. COVID-19 specific harm reduction recommendations messages were adopted by all ACS and disseminated to inpatients [10]. Messaging included keeping extra naloxone on hand or cleaning syringes with bleach when access to sterile needles were not available [10]. In Vancouver, innovative strategies such as virtual supervision methods which could call 911 to a pre-specified location should someone become unable to respond to prompts provided by their cell phone were developed to monitor for unintentional overdoses. If interested, patients were connected to these resources while hospitalized.

### Discharge planning adaptations

Discharge planning was particularly challenging early in the pandemic for people with SUD infected with COVID-19. Few community detoxification, sober homes, residential programs, and shelters accepted people who were COVID-19 positive, leading to higher rates of unsafe discharge plans (e.g. discharging to the street). At all sites temporary isolation and recovery unites were eventually established for people unable to safely isolate (e.g. person’s experiencing homelessness) to recover from COVID-19 and reduce transmission in the shelter systems, on the street, and within crowded housing. These units offered some patients with SUD a place to go following their acute care hospitalization. The establishment of the units required intensive ACS support to aid with the rapid development of SUD protocols to ensure withdrawal and SUD treatment in these units, care coordination for hospitalized patients, expansion of telemedicine consultations, coordination with opioid treatment programs to deliver methadone, and SUD care continuation post discharge.

At BMC peer mentors and social workers also increased discharge outreach for high-risk patients, in some cases supported by the hospital providing prepaid cell phones for patients without phones. Cell phones provided to patients were flip phones pre-loaded with 1500 min; capabilities included calling, texting and internet access. At OHSU, lack of patient phones was a common barrier to telemedicine follow up and outreach early in the pandemic; however, through ACS advocacy with payers, pre-paid phones were made available later in the pandemic. At YNHH and St. Paul’s resources for phones were not provided to patients which limited access to both inpatient and outpatient telemedicine.

### Financial impact

Despite the increased impact of COVID-19 on people with SUD, the pandemic’s financial impact to the health service sector has threatened financial stability of ACS teams. Inpatient ACS remain limited across the continent and historically have operated on extremely restricted or unstable budgets. YNHH and BMC ACS teams were asked to redeploy physician staff for COVID-19 initiatives, necessitating back up coverage or a reduction in ACS services. At YNHH, hiring freezes halted the filling of open positions. At OHSU and BMC, ACS expansion efforts have been significantly delayed or cancelled. This is despite a perceived surge of hospital SUD related admissions in all settings following the first wave of the pandemic. In the US, billing for services was also limited without in-person consultation in the early pandemic, so many ACS saw a temporary reduction in revenue.

### Recommendations for the future

Our experiences, across four hospitals in North America, highlight critical challenges to providing care to hospitalized people with SUD during the COVID-19 pandemic and responses to these challenges (Table 1). As efforts to maintain physical distancing and preserve personal protective equipment are likely to remain for months to come, we believe that hospitals must invest in telehealth infrastructure within the hospital and consider cellphone donations for people without cellphone access to help with post-discharge follow-up. We see such concrete steps to maintain access to care by integrating or providing telemedicine tools as critical components to providing equitable care to patients such as those with SUD experiencing homelessness who have limited access to technologies needed for telemedicine. BMC ACS is currently evaluating its cellphone distribution program. Further studies evaluating telehealth and other inclusive approaches to telecommunication technologies are needed to understand current gaps and potential impacts of telehealth, particularly with regards to vulnerable populations.

We believe that ACS were essential to hospitals’ readiness to support systematically marginalized patients during the pandemic. ACS supported SUD management and care coordination in COVID-19 isolation and recovery units and led regional SUD care coordination efforts. ACS further leveraged the interdisciplinary team approach during the pandemic where social work and peer services enhanced inpatient and post discharge outreach. Qualitative studies evaluating the impact of this active outreach strategies on the hospitalization experience would be helpful to gain a nuanced understanding of the pros and cons of more active outreach. Additionally, research evaluating the impact of contact with ACS during the pandemic on substance use and other health-related outcomes could quantify its role in supporting hospitalized people with SUD during public health crises. For example, we encourage other ACS leaders and addiction clinicians to track metrics that may be impacted by ACS, such as rates of discharges against medical advice among COVID-19 positive patients with SUD who connected with ACS versus those that did not.

Regulations loosened during the COVID-19 pandemic, for example tele-buprenorphine access in the US and safer supply programs in Canada, resulted in perceived increased engagement in care during hospitalization as some barriers preventing patients from accessing ongoing care were reduced. Due to The Controlled Substances Act, safe supply programs currently undergoing evaluation in Canada remain illegal in the US. While studies evaluating the impact of the regulatory changes to safe supply in Canada and MOUD in the US are in progress, we believe that, in the absence of evidence, diminishing barriers in the community to MOUD and harm reduction are critical and had positive impacts on the inpatient management experience.

## Conclusion

Despite regulatory changes and rapid adaptations, our hospital-based ACS teams were not able to compensate for the larger systemic gaps in the substance use treatment system that persisted and were undoubtedly exacerbated by COVID-19. Ongoing studies at our institutions and others will guide future evidenced based ACS adaptations, but while we wait for empirical data, we hope the experiences shared here, from four ACS impacted early in the COVID-19 pandemic, may serve as a roadmap for other areas who are more recently facing a surge of COVID-19 admissions. As the economic impact threatens the very existence of ACS, overdose deaths continue rising across North America, highlighting the essential nature of these services [3]. In light of these disturbing statistics, we believe that it is imperative for health care systems to continue investing in addiction care, including hospital-based ACS, during public health crises.

## Data Availability

Not applicable.

## Abbreviations

COVID-19: Novel Coronavirus Disease 2019
SUD: Substance use disorders
ACS: Addiction consult services
OHSU: Oregon Health & Sciences University
BMC: Boston Medical Center
YNHH: Yale New Haven Hospital
