# Correction to: Adapting inpatient addiction medicine consult services during the COVID‑19 pandemic

**DOI:** 10.1186/s13722-021-00232-y

**Published:** 2021-04-28

**Authors:** Miriam T. H. Harris, Alyssa Peterkin, Paxton Bach, Honora Englander, Emily Lapidus, Theresa Rolley, Melissa B. Weimer, Zoe M. Weinstein

**Affiliations:** 1 Grayken Center for Addiction, Boston Medical Center, Boston, MA USA; 2 Clinical Addiction Research and Education (CARE) Unit, Section of General Internal Medicine, Department of Medicine, Boston University School of Medicine and Boston Medical Center, 801 Massachusetts Avenue, Second Floor, Boston, MA 02118 USA; 3 British Columbia Centre On Substance Use, St. Paul’s Hospital, Vancouver, BC Canada; 4 Department of Medicine, University of British Columbia, Vancouver, BC Canada; 5 Division of Hospital Medicine, Department of Medicine, Oregon Health and Science University, Portland, OR USA; 6 Program in Addiction Medicine, Department of Medicine, Yale School of Medicine, New Haven, CT USA

## Correction to: Addict Sci Clin Pract (2021) 16:13 https://doi.org/10.1186/s13722-021-00221-1

In the original publication of this article [1], the author noticed that the title of Table 1 is incorrect and the format of Table 1 also has problem, the correct Table 1 is given below. The original publication has been corrected.

**Table 1:**
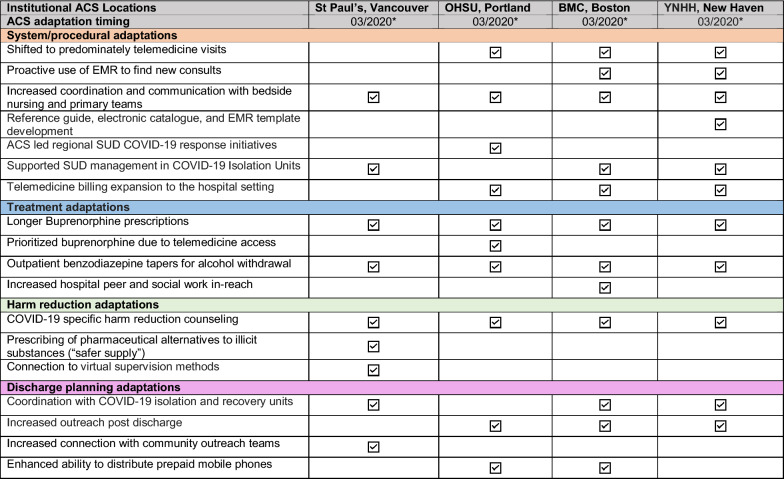
Addiction consult service COVID-19 adaptations

*COVID-19* Novel Coronavirus Disease 2019, *ACS* addiction consult service, *OHSU* Oregon Health & Sciences University, *BMC* Boston Medical Center, *YNHH* Yale New Haven, *EMR* electronic medical record, *SUD* substance use disorder

^*^Changes that were instituted starting in March have been dynamic based on local case rates and guidance from local hospital leadership and public health departments
